# Current Hospital Topics

**Published:** 1906-12-29

**Authors:** 


### HOSPITAL ADMINISTRATION.

### CONSTRUCTION AND ECONOMICS.

### CURRENT HOSPITAL TOPICS.

#### The Disinfection of Bedding and Clothes.

A fair number of hospitals have provided themselves with disinfectors, and several are at the present time contemplating their erection; but a more economical way of dealing with infected linen, etc., would be to obtain the sanction of the local corporations or Isolation Hospital Boards for the work to be done at the isolation hospitals. The disinfectors at these institutions are, generally speaking, constantly at work and could undertake voluntary hospital work without probably any further outlay beyond the mere cost of collection and delivery.

#### St. Mary's Hospital, Manchester.

The importance of this hospital has been much increased by the sanction given by the Charity Commissioners to the scheme by which it has been amalgamated with the Manchester Southern and Maternity Hospitals. It has been determined to erect a new hospital in High Street, Oxford Road, near Whitworth Park. On the completion of the new hospital, which will provide accommodation for 80 women and 25 children, or 105 beds in all, the old buildings in Whitworth Street, W., will be converted into an out-patients' department and Maternity Hospital, where a school will be established for students and midwives. The expense of the new buildings is to be defrayed by a gift of Mr. Edwin Reid, trustee of the late Manassah Gledhill, who has undertaken to provide £50,000, or so much of it as may be necessary, to defray the cost of the new hospital buildings. It is proposed to meet the outlay on equipment and furniture out of the ordinary funds of the joint hospitals.

#### An Amenity of Hospital Work.

A very interesting interchange in hospital life will shortly take place. As is well known, the recently amalgamated Royal and National Orthopædic Hospitals are engaged upon rebuilding, and in order to proceed with the work it has been found necessary to take temporary quarters for their patients. It is equally well known that several wards at Charing Cross Hospital are closed for want of funds, and the committee of the Royal National Orthopædic Hospital have arranged to rent a number of the empty beds at Charing Cross Hospital for the use of patients from the Orthopædic Hospital. The Royal National Orthopædic Hospital are to treat and nurse the patients, and the kitchens of Charing Cross are to be called upon to feed them. This arrangement will be brought into effect early in the new year. With beds at Charing Cross Hospital available and evidently suitable for the patients, the public may ask for evidence to show the necessity for providing them at the Royal National Orthopædic Hospital.

#### Income Tax and its Repayment.

It is a well-known fact that there is constantly in the hands of the Inland Revenue authorities a very large sum due to taxpayers who are entitled to refunds. A case of this where charities are the losers has recently come to light. The profits of the London Labourers' Dwellings Society, Limited, are by the articles of association payable to the charities nominated by the holders of the shares. For very many years the dividends have been paid less tax, but no mention of the fact has ever been made upon the dividend warrants, and doubtless the great majority of the recipients have never included the amount in their annual claim for refunds. It has been suggested to the Society that the warrants should follow the common form and so constitute a certificate of payment of income-tax in accordance with the custom of such undertakings. In any case the officials of the charities interested should obtain from the Secretary of the Society a certificate of payment of income-tax in the usual form, and then include the sum involved for the past three years in their next claim for repayment.

#### Birmingham General Hospital.

The hospital and conjoint Committees of the British Medical Association have laid down the principle that it is desirable that there shall be an age limit for the retirement of all medical officers. The best administered hospitals have long had such an age limit, and those governing bodies which have displayed the greatest intelligence have enforced this system of periodical election for honorary medical officers. The object is not only to secure efficient administration, but to maintain an active personal interest in the institution and the work on the part, alike, of the medical staff, the Governors and the public. The Birmingham General Hospital, like other well administered establishments, has an Election Committee, consisting of the members of the Board of Management, and of 50 Governors who are chosen by ballot at the annual meeting. At a meeting of this Committee just held, in accordance with the regulations, it was unanimously decided to reappoint the following honorary officers for a period of ten years: Dr. Robert M. Simon, Hon. Physician; Mr. Gilbert Barling, Hon. Surgeon; and Mr. William F. Haslam, Hon. Surgeon. As showing the work of this system it may be interesting to add, that Dr. Simon was appointed Assistant Physician in 1879, and Honorary Physician in April 1891. Mr. Gilbert Barling was elected Resident Registrar and Pathologist in February 1880, Resident Surgical Officer in August 1882, Assistant Surgeon in 1886, and Hon. Surgeon in June 1891.

Mr. Haslam was appointed Assistant Surgeon in February 1882, and Hon. Surgeon in 1891. There can be no doubt that the modern system of electing honorary medical officers now in force at the greater hospitals throughout the country secures full knowledge of the relative qualifications of the various candidates. The election is founded on a statement of services rendered to the institutions in the junior offices, and thus the selection is based upon merit resting upon knowledge, and so the best men secure the higher posts. This is all to the good, and the system has been strengthened by the higher standard of medical education which has entailed the selection of the most capable of the younger men to fill various paid posts on the teaching staffs of the hospitals.

